# A study on the DAM-EfficientNet hail rapid identification algorithm based on FY-4A_AGRI

**DOI:** 10.1038/s41598-024-54142-5

**Published:** 2024-02-12

**Authors:** Renfeng Liu, Haonan Dai, YingYing Chen, Hongxing Zhu, DaiHeng Wu, Hao Li, Dejun Li, Cheng Zhou

**Affiliations:** 1 School of Mathematics and Computer Science, Wuhan Polytechnic University, Wuhan, 430023 China; 2 Hubei Meteorological Service Center, Wuhan, 430205 China; 3 Guizhou Provincial Office of Artificial Weather Modification, Guiyang, 550081 China; 4 Hubei Key Laboratory of Intelligent Wireless Communications, South-Central Minzu University, Wuhan, 430074 China

**Keywords:** FY-4A, Hail, Deep learning, DAM-EfficientNet, Climate sciences, Climate change, Cryospheric science

## Abstract

Hail, a highly destructive weather phenomenon, necessitates critical identification and forecasting for the protection of human lives and properties. The identification and forecasting of hail are vital for ensuring human safety and safeguarding assets. This research proposes a deep learning algorithm named Dual Attention Module EfficientNet (DAM-EfficientNet), based on EfficientNet, for detecting hail weather conditions. DAM-EfficientNet was evaluated using FY-4A satellite imagery and real hail fall records, achieving an accuracy of 98.53% in hail detection, a 97.92% probability of detection, a false alarm rate of 2.08%, and a critical success index of 95.92%. DAM-EfficientNet outperforms existing deep learning models in terms of accuracy and detection capability, with fewer parameters and computational needs. The results validate DAM-EfficientNet’s effectiveness and superior performance in hail weather detection. Case studies indicate that the model can accurately forecast potential hail-affected areas and times. Overall, the DAM-EfficientNet model proves to be effective in identifying hail weather, offering robust support for weather disaster alerts and prevention. It holds promise for further enhancements and broader application across more data sources and meteorological parameters, thereby increasing the precision and timeliness of hail forecasting to combat hail disasters and boost public safety.

## Introduction

Hail, a weather event arising from intense updrafts, is notorious for intense rainfall and the formation of ice pellets. It significantly harms humans, agricultural fields^1–3^, and structures. Research by Martín Battaglia and others^4^ suggests that hail can cause leaf loss in plants, potentially decreasing yields by as much as 30%. In daily life, numerous vehicles parked outdoors are vulnerable to hail, with ice balls impacting and damaging the vehicles’ surfaces. The study by Roman Hohl et al.^5^ introduced a damage assessment function for cars affected by hail storms, correlating with the kinetic energy of hail measured via radar. Bell, J. R., et al.^6^ demonstrated the effectiveness of using satellite imagery for automatic detection of hail damage on crops. By utilizing NDVI and ground surface temperature data and applying threshold and anomaly detection methods, they successfully distinguished hail damage across different growth seasons, providing solid support for precise hail impact monitoring in the agricultural sector. Moreover, Heinz Jürgen Punge and his team^7^, through a 14-year satellite study, identified the key distribution zones and high-incidence periods for hail in South Africa, offering important data for long-term hail risk assessments. W.J.W. Botzen and associates^8^ pointed out the direct correlation between hail activities and agricultural losses, with future projections indicating an increase in hail-induced damage to open-air farming between 25 and 50%. These instances underscore the pressing need to address hail weather challenges both locally and globally. Meteorological researchers urgently require fast, effective, and convenient methods to accurately detect hail in cloud formations, aiding in the timely prevention of hail threats and minimizing human and financial losses.

In the last several decades, there has been notable advancement in the study of methods for identifying hail. Numerous scholars in meteorology have engaged in research to more deeply understand the features of hail clouds. One prevalent approach is the traditional study based on radar equipment data. In this vein, many researchers use radar’s differential reflectivity to determine the presence of hail. Pamela L. Heinselman and Alexander V. Ryzhkov^9^ streamlined the fuzzy logic hydrometeor classification algorithm (HCA), which utilizes four radar metrics: reflectivity, differential reflectivity, cross-correlation coefficient, and reflectivity texture. This algorithm effectively discerns between seven echo types, including rain and hail. The HCA’s ability to classify hail was validated using polarimetric radar and on-the-ground data, showing that it surpasses the Hail Detection Algorithm (HDA) in hail detection probability and accuracy. In early hail research, THOMAS A. SELIGA and team^10^ clearly and dependably separated hail from rain areas using differential reflectivity radar, based on the distinct polarization scattering characteristics of the two. In recent advancements in satellite technology, the study by Ralph Ferraro and others^11^ stands out. They developed an innovative probabilistic hail detection method using passive microwave measurements from satellites. This approach effectively distinguishes moderate and severe hail storms by analyzing the effect of hail on the brightness temperatures detected by satellites. This method has been validated in selected cases across Europe, South America, and the USA, showcasing its versatility and adaptability on various satellite sensors. Additionally, Pablo Melcón and colleagues^12^ have achieved significant progress in enhancing the precision of hail monitoring. Through field validation in the south of France, they demonstrated the efficacy of their newly developed hail detection tool (FHDT) based on the Meteosat Second Generation (MSG) satellite data, particularly in reducing false positives. These examples underscore the viability and significance of satellite data utilization in hail detection.

The rapid advancements in machine learning and artificial intelligence neural networks have led to their burgeoning application in hail detection research. David John Gagne et al.^13^ innovated a probabilistic machine learning method to advance hail forecasting, addressing the shortcomings of traditional techniques. This method, utilizing object detection and tracking algorithms, excels at pinpointing potential hailstorms, outperforming traditional approaches in the Critical Success Index (CSI). In related studies^14–16^, machine learning algorithms were used to identify hail weather using C-band radar echoes. Notably, models incorporating Support Vector Machine, Decision Tree, and Naive Bayes were compared, showing Probability of Detection (POD) and False Alarm Rate (FAR) values of 88.9% and 19.6%, 90.5% and 24.1%, and 67.8% and 10.6%, respectively. It was observed that a higher POD often correlates with a higher FAR, in line with prior research findings. Regarding deep learning, Melinda Pullman et al.^17^ in 2019 demonstrated the capability of deep learning networks in meteorological phenomenon recognition, focusing on hailstorm detection. In 2021, Lan et al.^18^ developed a dataset comprising 11 Doppler weather radars, regional automatic stations, lightning positioning data, and hail station records. They employed deep learning methods, specifically PredRNN++ and Resnet, for automated hail identification, achieving a high accuracy of 93.81%. Furthermore, in 2023, Stavros Kolios^19^ utilized a Deep Neural Network (DNN) model based on multispectral infrared images from the European Meteorological Satellite System’s (MTG) third generation, trained with extensive data from the European Severe Weather Database (ESWD), yielding highly satisfactory results.

When identifying hail using machine learning and artificial intelligence technologies, it is crucial to meticulously process and filter the data to ensure its high quality and reliability, thereby minimizing the impact of inaccuracies due to data errors. Kiel L. Ortega and others^20^ remotely collected hail data from severe thunderstorms at high temporal and spatial resolutions through the Severe Hail Analysis, Verification, and Evaluation (SHAVE) project. High-resolution data reports enhance the effectiveness of hail detection and warning methods. Scott F. Blair and colleagues^21^ compiled hail measurement data from 73 severe thunderstorms, providing a benchmark for exploring meteorological hail databases and hail forecasting in weather warning products. Therefore, further research still needs to focus on improving data quality to enhance the accuracy and reliability of data studies on weather phenomena like hail and other intense updrafts.

To enhance the early warning detection of hail in the atmosphere, there is a need for a more efficient method to identify hail clouds. This research, therefore, adopts a novel approach to data acquisition using the FY-4A satellite^22^. Leveraging meteorological data from the FY-4A satellite and performing preprocessing on the raw satellite data to generate cloud images, we have developed a novel lightweight deep learning model, referred to as DAM-EfficientNet (Dual Attention Module EfficientNet). This model is designed for small sample datasets and is capable of identifying features of hail clouds in FY-4A satellite cloud imagery, thereby presenting a new frontier for effective hail detection.

The objective of this research is to introduce a more efficient methodology for the timely detection and early warning of hail, aiming to reduce its potential impacts on human lives, agriculture, and transportation systems. Capitalizing on the advancements in meteorological sciences and artificial intelligence, this study leverages data from the FY-4A satellite along with the lightweight deep learning model DAM-EfficientNet. This innovative approach is designed to overcome the drawbacks of traditional methods, thereby enhancing the accuracy of detecting atmospheric hail clouds. By thoroughly analyzing satellite cloud imagery data, this research is committed to providing a more reliable tool for forecasting and mitigating the effects of hail, aiming to minimize human and economic losses associated with hail incidents. The study anticipates that its contributions will offer groundbreaking techniques in hail monitoring and prevention within the field of meteorology, thus providing more effective safeguards for society.

## Related works

### Dataset

**Figure 1:**
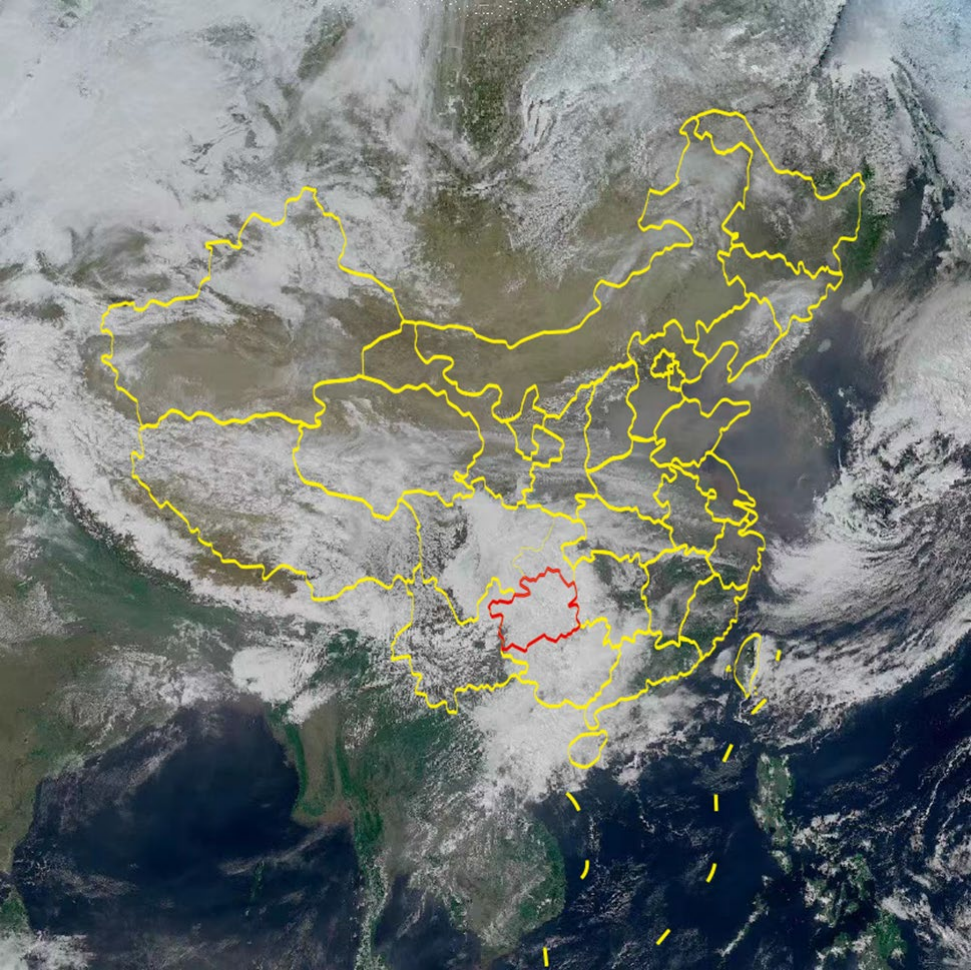
True-color image of China. *Note*: This image was generated using Python 3.8 with the aid of Satpy 0.29, Cartopy 0.18, Matplotlib 3.3.1, and Geopandas 0.8.1. For more details, visit: https://bit.ly/486tfYA.

In this study, we have comprehensively utilized FY-4A satellite data from 2019 to 2022, along with ground hail observation records from Guizhou Province, to conduct hail identification research. The FY-4A satellite, China’s latest generation of geostationary meteorological satellites, was launched on December 11, 2016. Initially positioned at 99.5^∘^E above the equator, it drifted to 104.7^∘^E on May 25, 2017. As the world’s first satellite combining geostationary orbit imaging observation with infrared hyperspectral atmospheric vertical detection, FY-4A is equipped with an advanced Advanced Baseline Imager (ABI) as its primary payload. ABI, with its precise dual-scanning mirror mechanism, achieves accurate and flexible two-dimensional targeting and rapid scanning of designated areas. Its off-axis, three-mirror primary optical system boasts a high-frequency acquisition capability of earth cloud images over more than 14 spectral bands and utilizes onboard blackbody for high-frequency infrared calibration, ensuring the precision of observational data. This imager is primarily used for cloud imaging, capturing aerosols, snow, and water vapor clouds of different phases and altitudes. Figure 1 in this document showcases a comprehensive true-color representation of the China area, generated from the visible light channels aboard the FY-4A satellite. These images, aligning closely with how the human eye perceives natural hues, offer a direct visual insight into China’s natural landscapes. The image highlights China’s borders in yellow, with Guizhou Province delineated in red. As a focal point for observing hail events in this research, Guizhou’s climatic patterns and geographical features are of paramount importance. The satellite’s infrared and microwave sensors also play a crucial role, providing essential details about the cloud tops’ temperature and their vertical structure, crucial for spotting cumulonimbus clouds likely to produce hail, making it possible to more effectively monitor and analyze hail-related weather patterns by combining these true-color visuals with infrared and microwave data.

The detailed documentation of ground-level hail occurrences, along with data from the FY-4A satellite, was provided by the Hubei Provincial Meteorological Service Center, associated with the corresponding author, and the Guizhou Provincial Office for Artificial Weather Influence involved in this study. These data sets, collected by well-trained observers, include detailed records of the aftermath and specifics of each hail event. The recorded hailstones had maximum diameters ranging from 2 to 30 mm, with an average maximum diameter of 8.3 mm. Importantly, approximately 76% of the total samples consisted of hailstones with diameters equal to or greater than 5 mm. These in-depth measurements provide a robust basis for our analysis, thereby improving the reliability and applicability of our research outcomes. In this paper, a dataset of hail events was constructed, detailing the specific locations, dates, and exact start and end times of each occurrence. The distinctive terrain of Guizhou Province, including its steep mountains, extensive hills, and broad basins, offers an optimal environment for the formation of intense convective airflows. This not only leads to the variability of local weather but also facilitates hail formation. In this geographic and climatic setting, our study meticulously examined 172 hail events that took place from 2019 to 2022 within the coordinates of 25^∘^08’N to 29^∘^04’N and 104^∘^66’E to 108^∘^92’E. These events encompassed 146 distinct regions within Guizhou Province. The analysis of this data is vital for understanding the specific characteristics and trends of hail formation in the region, and it also serves as a scientific foundation for future meteorological forecasting and disaster prevention strategies.

The FY-4A satellite is equipped with the Advanced Geostationary Radiation Imager (AGRI), comprising 14 observation channels, including two visible light channels, two near-infrared channels, and eight infrared channels. Notably, the infrared channels, specifically channels 7 to 14, facilitate continuous observation both day and night. These channels, with a spatial resolution of 2 to 4 kilometers, enable the satellite to complete a scan of the entire Chinese territory in just five minutes, significantly faster than the traditional 15-minute full-disk image scan. This rapid scanning capability is crucial for capturing subtle changes in hail features. Detailed specifications of the satellite’s performance parameters are listed in Table 1, encompassing a variety of channels such as visible light and near-infrared, short-wave infrared, mid-wave infrared, water vapor, and long-wave infrared, each tailored for specific observational purposes and applications. International studies^23–26^ have examined infrared imager wavelengths and channels in geostationary satellites. Previous research^27^ indicates that infrared imagers with a central wavelength of 3.75$\upmu$m are suitable for cloud characteristic analysis, hotspot detection, and snow identification. In this study, Channel 7 was specifically chosen as the primary observation channel. As hail typically forms in cooler cloud top regions, the mid-wave infrared channel is instrumental in locating potential hail formation areas. Additionally, since hail formation is often associated with the development of convective clouds, using data from Channel 7 allows for more accurate cloud top identification, thus enhancing the precision of hail localization. The 2-kilometer spatial resolution of this channel provides sufficient detail to differentiate between cloud characteristics and potential hail formation areas in different regions, laying a solid foundation for building a high-quality hail dataset.

**Table 1:** The performance parameters of FY-4A satellite.

Channel	Channel type	Center wavelength $\upmu$m	Spatial resolution (km)	Primary usage
1	Visible and near-infrared	0.47	1	True-color synthesis
2	Visible and near-infrared	0.65	0.5−1	Image navigation and registration
3	Visible and near-infrared	0.825	1	Aerosols over water surfaces
4	Shortwave infrared	1.375	2	Cirrus clouds
5	Shortwave infrared	1.61	2	Low clouds, Water clouds
6	Shortwave infrared	2.25	2−4	Cirrus clouds, Aerosols
7	Mediumwave infrared	3.75	2	Cloud top temperature
8	Mediumwave infrared	3.75	4	Surface
9	Water vapor	6.25	4	Upper-level water vapor
10	Water vapor	7.1	4	Mid-level water vapor
11	Longwave infrared	8.5	4	Total water vapor, Clouds
12	Longwave infrared	10.7	4	Surface temperature
13	Longwave infrared	12.0	4	Total water vapor amount
14	Longwave infrared	13.5	4	Water vapor

### Data preprocessing

In order to maintain consistency in time and space within the data, this research rigorously handled the time alignment between satellite observation data and on-ground actual records. The FY-4A satellite’s multi-channel scanning imaging data utilize Coordinated Universal Time (UTC) for timestamping, while the recorded hail events from ground observations follow Beijing Time (UTC+8). Therefore, for the synchronization of these two data sets, an adjustment of adding 8 hours was made to the satellite data timestamps to match them with Beijing Time.

Given the sparse nature of ground-based data on hail events, this study focused on the variation in cloud features observed by the satellite within the hour leading up to a hail event, using these as essential indicators for the deep learning model’s training. Considering the FY-4A satellite’s capability to conduct a complete scan of the China area every five minutes, the research used a time-point comparative analysis to ensure the precise correlation of features extracted from satellite images with ground observations.

In the data annotation process, this research implemented a rigorous synchronization protocol for time and space, guaranteeing the alignment of satellite imagery with ground observation records. A spatial window with a 5-kilometer radius was established as the standard for spatial precision in hail event identification. Time stamps and geographic coordinates of each satellite image file were precisely matched with ground observations, affirming the tight correlation of satellite-captured cloud features with the actual hail occurrence sites. For example, if hail was observed at a certain location from 8:00 to 8:30 on a specific date, satellite data from the hour preceding 8:30 were annotated as ‘hail present’. Similarly, data files were marked as ‘no hail’ based on accurate no-hail occurrence times and locations provided by the corresponding author. This approach ensures the scientific integrity and applicability of the annotated data, offering valuable training and validation datasets for the deep learning model.

Further, this study extracted images of hail occurrence areas based on satellite files, geographic coordinates, and actual data records. The specific steps included converting geographic coordinates into pixel coordinates and cropping the area images to a resolution of 256x256 based on a predetermined offset. Subsequently, the collection range was limited to within 4 kilometers, and the corresponding geographic coordinates were converted into a two-dimensional array, forming a 2D image suitable for model learning. Each image was then appropriately labeled according to its category (’hail present’ or ’no hail’). In order to develop a dataset with a consistent format, every image gathered was scaled to a uniform size of 224x224 pixels. A thorough examination of each image’s clarity and feature distinctiveness was conducted to guarantee the high quality of the sample collection.

**Figure 2:**
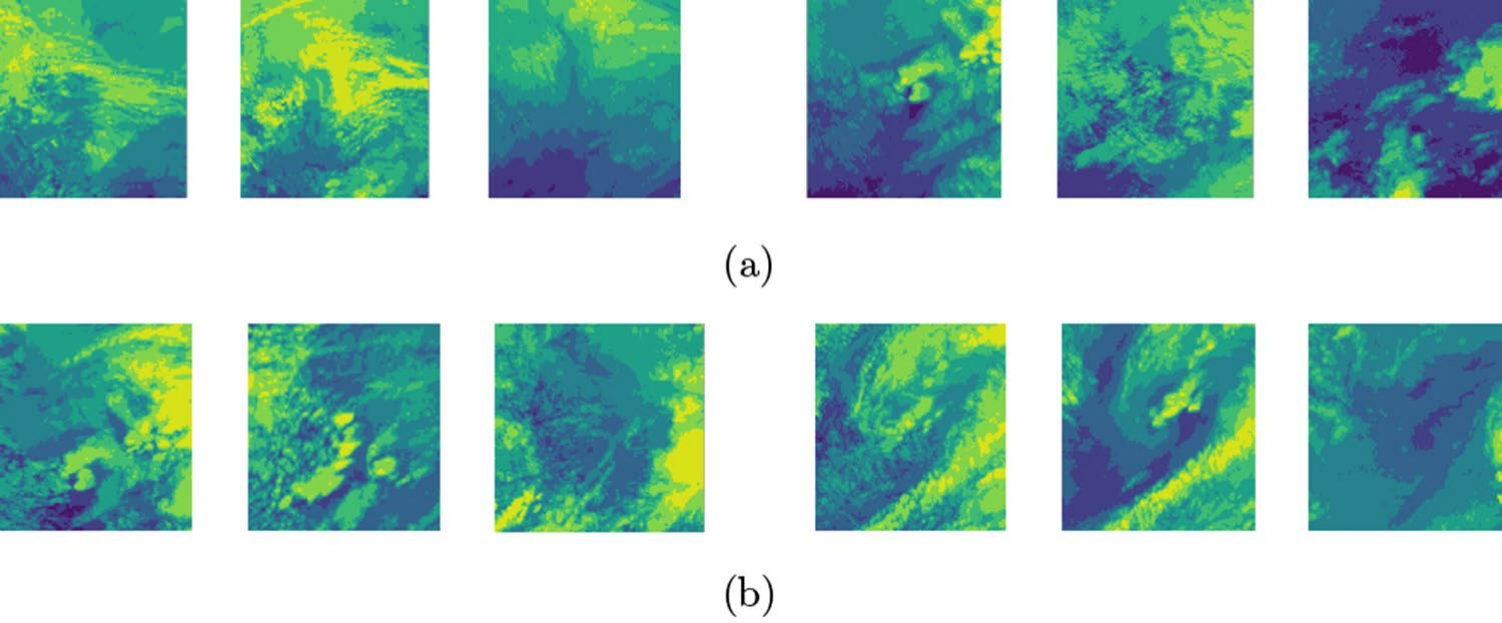
Example Images of Hail Sample Images (**a**) isHail Sample, (**b**) noHail Sample.

Following data preprocessing, our study has developed a hail dataset essential for deep learning model training. Illustrated in Fig. 2 are the curated image samples of hail occurrences versus non-hail scenarios, crafted not from raw satellite imagery but through a refined process extracting crucial information. Each image pixel equates to a 4 km ground area, facilitating an understanding of the depicted spatial range. Instead of mirroring cloud top temperatures directly, the color variations in these images encapsulate key hail-related data. Such delicate color variations, challenging for the naked eye to discern, are analyzed and recognized through Convolutional Neural Networks (CNN). By employing its layered structure and convolutional techniques, CNNs adeptly extract various image features, including but not limited to color, shape, and texture, which might not be overtly noticeable. The training of our deep learning models enables CNNs to unearth intricate details pertinent to hail phenomena, underscoring their potent capability in identifying slight visual distinctions.

The dataset used in this study comprises two distinct parts: the training set and the testing set, encompassing a total of 681 images. For the construction of the training set, 80% of the images from each class were randomly selected, while the remaining 20% were assigned to the testing set. The training set consists of 545 images, whereas the testing set encompasses 136 images. Further details regarding the dataset’s composition are provided in Table 2.

**Table 2:** The number of each type of image on the training and test dataset.

Data Categories	Train	Test
isHail	193	48
noHail	352	88

Due to the limited availability of FY-4A satellite data and real hail records, this study adopts the lightweight and high-precision EfficientNet model as the baseline network.

### EfficientNet

EfficientNet^28^ is an efficient convolutional neural network architecture proposed by the Google Brain team in 2019. It optimizes the relationship between three core factors in convolutional neural networks: network depth, network width, and resolution, achieving improved performance with reduced computational resources. Unlike traditional convolutional neural networks that typically enhance accuracy by adjusting individual dimensions of network depth, input resolution, or convolutional channel number, EfficientNet introduces an innovative approach. It first identifies a set of optimal hyperparameters for the network structure through random search and then explores increasing depth, width, and resolution based on this foundation. The authors introduced a compound scaling method that uniformly scales the width, depth, and resolution parameters using a compound factor $\phi$. The specific calculation formula is as follows:

1 $$\begin{aligned} {\left\{ \begin{array}{ll}d=\alpha ^\phi \\ w=\beta ^\phi \\ r=\gamma ^\phi \\ s.t.\alpha \cdot \beta ^2\cdot \gamma ^2 \approx 2 \\ \alpha \ge 1, \beta \ge 1, \gamma \ge 1 \end{array}\right. } \end{aligned}$$

In the formula, $\alpha$,$\beta$, and $\gamma$ represent constants that determine how different hyperparameters are allocated to the network’s depth, width, and image resolution.$\phi$ is the compound coefficient used to control model expansion. “s.t.” represents the constraint condition.

The compound scaling method is as follows: Using the Neural Architecture Search (NAS) method on the base network EfficientNet-B0 to search for $\alpha$, $\beta$, and $\gamma$, first, $\phi$ is fixed at 1, and the values of $\alpha$,$\beta$, and $\gamma$ are determined through grid search. Then, with $\alpha$,$\beta$, and $\gamma$ fixed as constants, an appropriate set of scaling factors $\phi$ is searched, resulting in EfficientNet-B0 to EfficientNet-B7.

EfficientNet^28^ provides a total of 8 different models from B0 to B7, enabling the selection of the appropriate model based on specific task requirements and computational resources. Furthermore, the EfficientNet^29^ model can be combined with traditional models like ResNet^30^, MobileNet^31^, etc., to further enhance model performance.

After experimenting with the dataset, it was found that an image resolution of 256$\times$256 achieves optimal results. To choose the best model as the base network for the hail identification task, this study considered both the model’s recognition performance and parameter size. Ultimately, EfficientNet-B1 was selected as the base network, using a specific set of network parameters ($\phi$=1, $\alpha$=1.2, $\beta$=1.1, $\gamma$=1.15) for further work.

## Approach

### Hail clouds and channel selection for FY-4A

In this study, deep learning technology is applied to analyze FY-4A satellite data, specifically focusing on channel 7 (3.5 4$\upmu$m) of the Fengyun-4 satellite. Spectral characteristic analysis shows that the radiation values of channel 7 are highly sensitive to the scale distribution, shape, and thermodynamic phase (water and ice) of particles near the cloud top. This sensitivity makes the channel particularly suitable for identifying larger, ice-phase particles, such as hail. Researchers worldwide have utilized similar channel data from satellites like NOAA to monitor and identify hail clouds by setting thresholds or establishing specific indicators. Drawing on these studies, our model particularly utilizes the data from channel 7 of the FY-4A satellite for in-depth analysis and identification of hail clouds. These data are integrated into a deep learning model to enhance the monitoring and identification capabilities for hail events, especially in convective active areas.

Utilizing Convolutional Neural Networks (CNNs), this study conducts an in-depth analysis of FY-4A satellite data, effectively identifying hail events. Convection in the atmosphere, as a primary means of vertical movement of heat and moisture, has a critical impact on hail formation. The CNN focuses on analyzing changes in cloud top temperature and morphology in the satellite observational data, which often indicate potential hail formation. These changes reveal the vertical development of clouds during the convective process, providing important clues for identifying hail events.

Updrafts play a crucial role in the formation of hail. The model indirectly detects the presence of updrafts by analyzing the vertical thickness of clouds and their dynamic changes. Specifically, the model can identify clouds with significant updraft characteristics, such as rapidly developing cumulonimbus clouds, thereby identifying potential hail events.

The deep learning model used in this study does not directly simulate complex meteorological processes but instead identifies features related to these processes indirectly by learning and analyzing patterns in satellite images. The model, through analyzing a large amount of historical data, learns cloud patterns closely associated with hail formation. These features, closely related to meteorological concepts, enable the model to exhibit excellent capability in handling complex meteorological data.

Although deep learning technology excels in analyzing complex meteorological data, understanding and interpreting the model’s output still relies on basic knowledge of meteorology and physics. By combining these concepts with advanced deep learning methods, this study can more accurately interpret the model’s predictive results and provide a scientific basis for a deeper understanding of the physical processes of hail formation.

### CBAM

**Figure 3:**
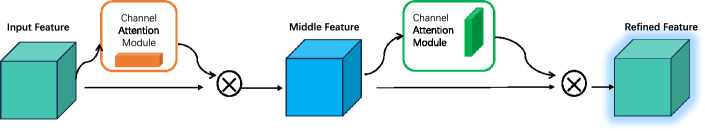
Channel attention and spatial attention in CBAM module.

CBAM (Cost Benefit Analysis Method)^32^ is a simple yet effective feed-forward convolutional neural network attention module proposed by Woo et al. in 2018. In contrast to the SE(Squeeze-and-Excitation) attention mechanism in EfficientNet, CBAM integrates channel and spatial attention mechanisms together, facilitating simultaneous consideration of inter-channel correlations and spatial details. The spatial attention mechanism takes into account the importance of different positions in the feature map when calculating attention weights, which helps the model achieve better performance in handling spatial information in images. Moreover, amidst a large amount of information features, CBAM focuses attention on the most relevant information for the current task, thereby suppressing unnecessary features. The internal structure of the CBAM module first utilizes the channel attention module and then applies the spatial attention module, as shown in Fig. 3.

**Figure 4:**
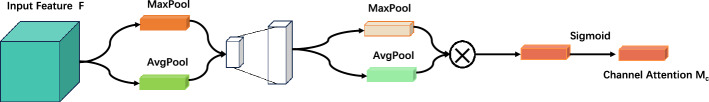
Channel attention module.

By applying channel attention, the input feature map dynamically learns the importance weight of each feature channel. This process involves computing the weight for each feature channel to capture the crucial information of the input features more effectively and outputting the optimized intermediate feature map. The channel attention module is illustrated in Fig. 4.

Assuming the input feature map F undergoes two parallel MaxPool layers and AvgPool layers, transforming the features from C$\times$H$\times$W to C$\times$1$\times$1 in size, followed by a shared Multilayer Perceptron (MLP) module. In this module, the channel dimension is first compressed to 1/r times its original size, then expanded back to the original channel size, and finally passed through the ReLU activation function to obtain two activated results. These two output results are element-wise added, and the merged feature vector is then mapped to [0,1] using a sigmoid activation function, resulting in the Channel Attention . The calculation process is given by:

2 $$\begin{aligned} M_c(F) =\sigma (MLP(Avgpool(F))+MLP(MaxPol(F))) \end{aligned}$$

where

$\sigma$ represents the sigmoid activation function;

MLP represents the Multilayer Perceptron module;

AvgPool denotes global average pooling;

MaxPool denotes global max pooling.

The obtained output $M_c$ is element-wise multiplied with the input feature map F, resulting in the adjusted intermediate feature map $F^{'}$ with channel attention. The calculation process is given by:

3 $$\begin{aligned} F^{'} = M_c(F)\bigotimes F \end{aligned}$$

where $\bigotimes$denotes element-wise multiplication; $F^{'}$represents the intermediate feature map outputted by the channel attention submodule.

In this study, we enhanced the spatial attention mechanism by replacing the global average pooling and global max pooling operations used in the original spatial attention module. While these operations effectively preserve the texture and background information of the image, they inevitably lead to the loss of some meaningful features. To address this, we employed 1$\times$1, 3$\times$3, and 5$\times$5 convolutions to replace the max pooling and average pooling^33^. Different convolution kernel sizes enable the capture of features with varying receptive fields: larger kernels extract more global features, whereas smaller kernels capture more local features.

The feature map $F^{'}$ of this module undergoes three convolution operations, resulting in the formation of two [1, W, H] weight vectors. These two feature maps are stacked using the Concat method, yielding a [2, H, W] spatial weight feature map. A subsequent 7x7 convolution operation is applied to reduce it to [1, H, W]. The resulting single-channel feature map represents the importance of each point on the feature map, where higher values indicate greater significance.

Next, the spatial weight [1, W, H] is element-wise multiplied with the original feature map [C, H, W], assigning weights to each point on the [H, W] feature map. This process is followed by applying the Sigmoid function to generate the final spatial attention feature map $M_s$. The expression for$M_s$is as follows:

4 $$\begin{aligned} M_S(F^{'}) = \sigma (f^{7\times 7}([f^{1\times 1}(F^{'});f^{3\times 3}(F^{'});f^{5\times 5}(F^{'})])) \end{aligned}$$

where $f^{7\times 7}$represents a 7$\times$7 convolution kernel.

**Figure 5:**
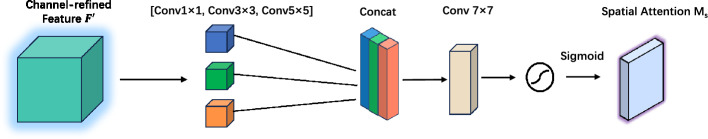
Spatial attention module.

The spatial attention module complements the channel attention module, enhancing the model’s recognition and localization of important targets based on their importance at different locations. The spatial attention module is illustrated in Fig. 5.

Finally, the spatial attention weight result $M_s$ is element-wise multiplied with the channel attention output result $F^{'}$ to obtain the CBAM output result $F^{''}$, as given by:

5 $$\begin{aligned} F^{''} = M_s(F^{'})\bigotimes F^{'} \end{aligned}$$

$F^{''}$represents the optimized feature map, which has been refined by the channel attention module and spatial attention module, enhancing the model’s capacity to learn key features in the image. This attention mechanism aids the model in effectively focusing on important regions in the spatial domain and suppressing unnecessary features, thereby improving the model’s recognition capability.

### ECA

**Figure 6:**
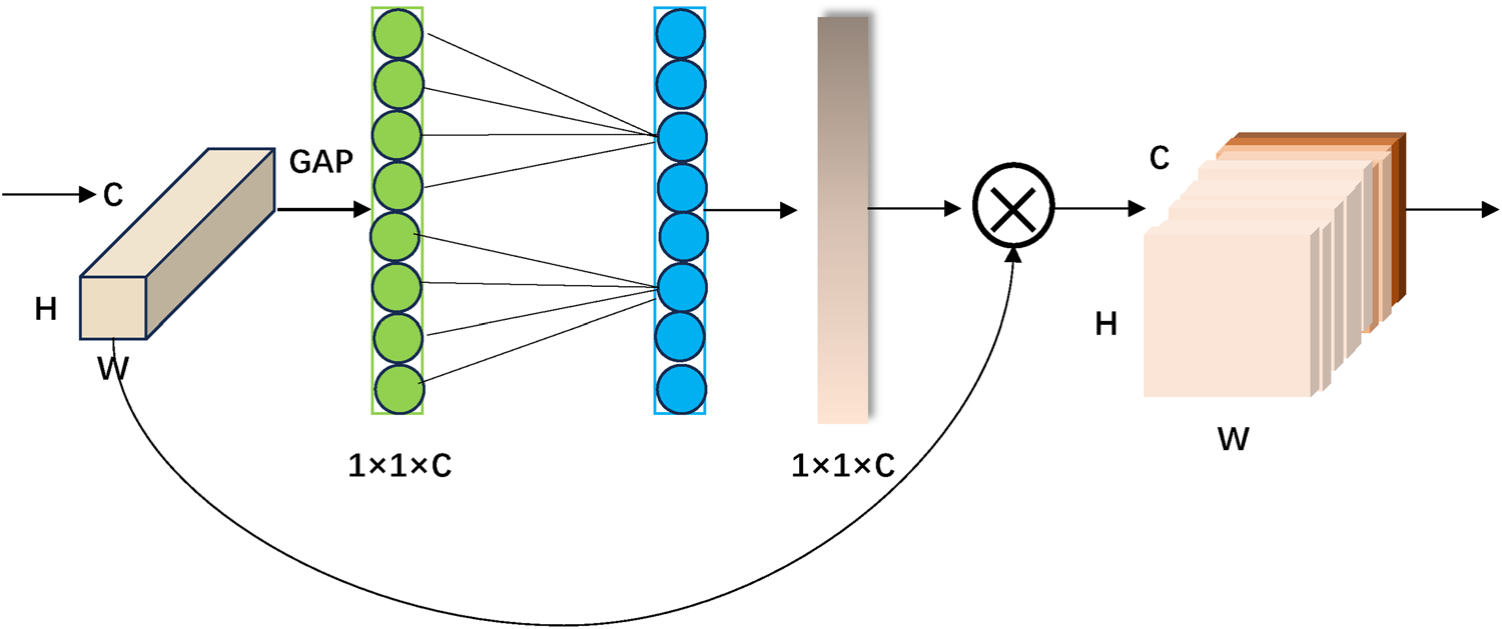
Structure diagram of the ECA module.

The ECA (Efficient Channel Attention) module^34^ is an efficient channel attention module. It employs a non-reducing local cross-channel interaction strategy and an adaptive selection of one-dimensional convolution kernel size for attention mechanism. The ECA attention mechanism module directly utilizes a 1x1 convolutional layer after the global average pooling layer to replace the fully connected layer. This module avoids dimension reduction while effectively capturing cross-channel interactions. In comparison to the SE block^35^, the ECA module does not reduce channel dimensions after global average pooling. Instead, it considers each channel and its k neighbors for local cross-channel interaction information. The schematic diagram of the ECA module is depicted in Fig. 6.

After applying global average pooling to the input feature map, the channel attention is generated using one-dimensional convolution. The adaptive one-dimensional convolution kernel size, k, is calculated based on the number of channels, C, in the feature map. Additionally, k represents the coverage of local cross-channel interaction, indicating that k neighbors participate in predicting the attention for each channel. The value of k is utilized in the one-dimensional convolution to derive the weights for each channel in the feature map. Finally, the normalized weights are element-wise multiplied with the original input feature map, resulting in the weighted feature map. The formula for the one-dimensional convolution kernel size is expressed as:

6 $$\begin{aligned} k = \mid \frac{log_{2}(C)}{y} +\frac{b}{y} \mid \end{aligned}$$

where C represents the number of channels;$y = 2,b = 1$ are utilized to adjust the proportion between the number of channels C and the size of the convolution kernel.

### DAM-EfficientNet

The EfficientNet baseline network consists of lightweight inverted bottleneck convolutional layers (Mobile Inverted Bottleneck Convolution, MBConv), each incorporating an SE (Squeeze-and-Excitation) module^36^. The SE module introduces attention mechanism in the channel dimension, allowing the network to automatically learn the importance of each channel in the feature map. It assigns a weight to each feature based on its importance, thereby enabling the neural network to focus on specific feature channels, enhancing those relevant to the current model, while suppressing less useful channels. However, the SE module only considers channel-wise information encoding and disregards positional information, which is crucial for recognition tasks, thus leading to some impact on the model’s performance in hail identification.

**Figure 7:**
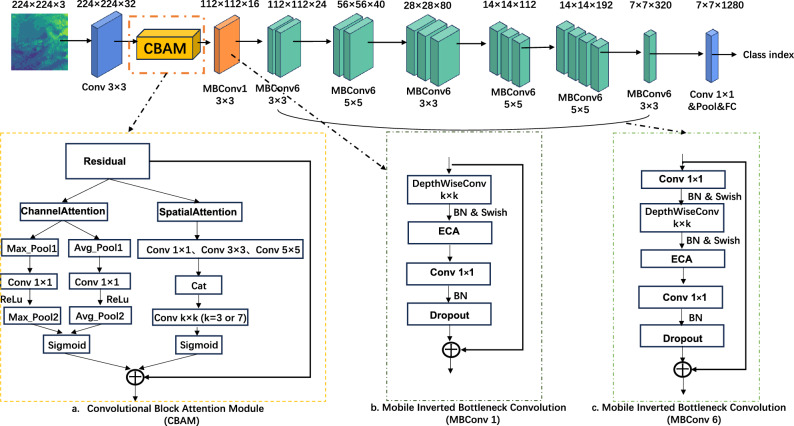
Structure diagram of DAM-EfficientNet. *Note*: ECA represents Efficient Channel Attention; CBAM represents Convolutional Block Attention Module; Conv represents convolution; Avg Pool represents average pooling; Max_Avg represents maximum pooling; MBConv represents Mobile Inverted Bottleneck Convolution; ReLu, Sigmoid, and Swish represent non-linear activation functions; k represents the convolution kernel size; Concat represents concatenation; BN represents batch normalization; $\oplus$ represents channel-wise addition; Dropout represents randomly deactivating nodes.

To further enhance the accuracy of the hail image recognition model, this study introduces the ECA (Efficient Channel Attention) and CBAM (Convolutional Block Attention Module) to develop the new DAM-EfficientNet model, aiming to improve the learning of attention mechanisms in hail visual recognition tasks. The DAM-EfficientNet network structure is illustrated in Fig. 7. In comparison to the baseline EfficientNet model, the following enhancements were made in this research:

1. The CBAM module is integrated in a residual structure after the first convolutional layer of the network. The CBAM module enables adaptive learning of spatial and channel attention weights in the image. This means the model can prioritize important regions and channels in the image, reducing computation on less significant areas. As a result, the model can more accurately extract key information with stronger expressive power, enhancing the global attention of the CNN. The network is less likely to lose crucial information due to previous convolutional operations, thereby improving the model’s ability to distinguish hail images.
2. The SE module in each MBConv module of the original EfficientNet network is replaced with the ECA module. The ECA module adjusts the weights of each channel, effectively capturing and enhancing useful information, thus improving the discriminative ability of features. This reduction in computation allows for more focused transmission of useful information, thereby enhancing the model’s recognition capability.

As depicted in Fig. 7, the hail classification process of DAM-EfficientNet is as follows: Firstly, any input hail image is preprocessed and transformed into a 224$\times$ 224 pixels $\times$ 3 channel image, which is then fed into the model. The feature map after the first convolution operation and the attention feature map enhanced by the CBAM module are multiplied using a residual approach to obtain a feature map with attention information. Subsequently, the hail image’s features are further extracted through 7 lightweight inverted bottleneck convolutional layers embedded with ECA modules, resulting in a 7$\times$7 pixels $\times$ 1280 channel feature map. Finally, the hail image recognition result is obtained through the fully connected layer.

## Experiments and analysis

### Experimental environment and parameter settings

In this experiment, the AMD Ryzen 7 4800H processor with a clock frequency of 2.6GHz and the NVIDIA GeForce GTX 2060 graphics card with 6GB of memory were used. The experimental device was equipped with 16GB of memory and ran on the Windows 10 operating system. The deep learning network models were implemented using the PyTorch 2.0.1 framework, with CUDA version 11.0. The experimental parameters for all network models were set as follows: the number of training epochs was fixed at 300, and each input sample had a batch size of 16. The model training process for hail recognition algorithm employed the Adam optimizer^37^ to update parameters, with an initial learning rate of 0.0001, and exponential decay rates $\beta _1$ and $\beta _2$ set to 0.9 and 0.999, respectively. To meet the constraints imposed by the hail recognition algorithm experiment, and to achieve a balance between accuracy and speed in hail recognition, the model’s input image size was set to 224$\times$224.

### Validation and evaluation metrics

In this study, we will use the same dataset for both training and testing the model through cross-validation. We will calculate various quantitative evaluation metrics, including accuracy (ACC), probability of detection (POD), false alarm rate (FAR), and critical success index (CSI), using the confusion matrix.

The accuracy (ACC) metric represents the proportion of correct predictions made by the classification model over the total number of observations and is computed as follows:

7 $$\begin{aligned} \mathrm {Accuracy = \frac{TP+TN}{TP+TN+FP+FN}} \end{aligned}$$

The probability of detection (POD) metric indicates the ratio of correctly classified hail events to the total number of observed hail events, reflecting the frequency of hail detection when hail is present. If all test images containing hail are correctly classified, POD will be equal to 1. On the other hand, if all test images containing hail are incorrectly classified, POD will be equal to 0. The calculation for POD is as follows:

8 $$\begin{aligned} \mathrm {POD = \frac{TP}{TP+FN}} \end{aligned}$$

The false alarm rate (FAR) metric represents the ratio of incorrectly classified non-hail events to the total number of predicted hail events. In other words, FAR is a measure of the network’s reliability, and a lower FAR value indicates fewer false alarms. The calculation for FAR is as follows:

9 $$\begin{aligned} \mathrm {FAR = \frac{FP}{TP+FP}} \end{aligned}$$

The critical success index (CSI) metric combines POD and FAR into a single score and represents the confidence level of the trained network in detecting hailstorms. CSI is calculated as follows:

10 $$\begin{aligned} \mathrm {CSI = \frac{TP}{TP+FP+FN}} \end{aligned}$$

where TP, FP, FN, and TN represent true positive, false positive, false negative, and true negative, respectively.

### Ablation study

In this study, the DAM-EfficientNet model was trained using the aforementioned experimental settings. The experimental dataset consisted of 241 images with hail and 440 images without hail, totaling 681 images. Among these, 545 images were used for training, and 136 images were used for testing. During each training epoch, the results were recorded, and recognition metrics, including accuracy (ACC), probability of detection (POD), false alarm rate (FAR), and critical success index (CSI), were calculated using the confusion matrix.

At the end of each training epoch, the best model was saved locally. Subsequent parameters were compared with those of the best model, and if better parameters were found, they were replaced; otherwise, they remained unchanged. This process resulted in the final optimal model parameters. Through this training process, the DAM-EfficientNet model was progressively optimized and achieved excellent performance on the test set. These experimental designs and training strategies contribute to ensuring a reliable and high-performance recognition model.

DAM-EfficientNet is an improvement over the EfficientNet baseline model. To evaluate the effectiveness of different modules in the proposed approach, five ablation experiments were conducted to test the evaluation metrics of different methods on the baseline model. Firstly, the evaluation metrics of the baseline model were tested. Next, the Adam optimization algorithm was employed on the basis of the EfficientNet baseline model. Adam is an adaptive learning rate optimization algorithm that automatically adjusts the learning rate based on the gradients of different parameters, thereby enhancing model convergence speed and performance. Additionally, the CBAM module was introduced and combined with a residual structure on the basis of the baseline network. The CBAM module is based on channel and spatial attention mechanisms, enabling the network to automatically learn and focus on important feature information, thereby enhancing the model’s representation capability. The introduction of the residual structure helps the network better capture feature and gradient information, enhancing information transmission and learning ability.

Furthermore, the attention mechanism in the backbone network was replaced with the ECA (Efficient Channel Attention) attention module, and Adam optimization was applied. The ECA attention module focuses on the feature response relationship between channels, further improving the attention to different channel features, and enhancing representation capability and performance. Finally, the Adam optimization algorithm, CBAM attention module, and ECA attention residual module were combined in the baseline model to obtain the final DAM-EfficientNet network model, which yielded excellent results. Through these five ablation operations, the DAM-EfficientNet model was improved and optimized to enhance its performance and accuracy. These improvement strategies help the model better learn important features and optimize global parameters through optimization algorithms, resulting in better results.

According to the data in Table 3, when the EfficientNet-B1 model was applied to the hail dataset, the model’s accuracy, POD, FAR, and CSI were 93.38%, 91.67%, 10.20%, and 83.02%, respectively. To further improve accuracy, the baseline model’s SGD optimizer was replaced with the Adam optimizer. The results showed that accuracy increased by 0.74%, CSI increased by 2.43%, and POD increased by 6.24%. Compared to the baseline model, the hit rate for correctly classifying hail significantly improved. From the data comparison, it can be observed that using the Adam optimizer for model optimization is more effective than using the SGD optimizer. However, after adding the Adam optimizer, the FAR increased by 2.76%, indicating a slight impact on predicting non-hail data. As for the improved version EfficientNet-B1-ECA of the EfficientNet-B1 model, the ECA attention mechanism was added in the backbone network to obtain better attention effects than the original network modules. Compared to the baseline network EfficientNet-B1, EfficientNet-B1-ECA improved accuracy, POD, and CSI by 2.21%, 4.16%, and 3.01%, respectively. Compared to the experiment with the Adam optimizer, the experiment with the ECA attention module reduced the FAR by 2.2%, indicating that the addition of the ECA module was more effective in recognizing non-hail data. Additionally, according to the data of the EfficientNet-B1-CBAM module in Table 3, adding the CBAM attention module with residual blocks to the original model increased accuracy from 93.38% to 97.06%, CSI increased from 85.46% to 91.84%, POD slightly increased by 2.08%, and the FAR decreased from 10.20% to 2.17%. This was a significant improvement over the baseline model, demonstrating the effectiveness of adding the CBAM module after the first module. The combination of channel attention mechanism and spatial attention mechanism in CBAM can improve the model’s feature representation ability and further reduce redundant information, enabling the model to focus more accurately on image content. Finally, when applying the proposed DAM-EfficientNet model, accuracy and CSI improved to 98.53% and 95.92%, respectively, and the FAR decreased to 2.08% from 10.20% in the initial baseline model. This indicates a significant improvement over the baseline model. These results demonstrate the feasibility and effectiveness of the proposed model improvement and network training strategies.

In conclusion, the DAM-EfficientNet model is a lightweight deep network model with excellent recognition accuracy.

**Table 3:** Ablation study for hail identification.

Model	Accuracy(%)	POD(%)	FAR(%)	CSI(%)
EfficientNet-B1	93.38	91.67	10.20	83.02
EfficientNet-B1-Adam	94.12	97.91	12.96	85.45
EfficientNet-B1-ECA	95.59	95.83	8.00	88.46
EfficientNet-B1-CBAM	97.06	93.75	2.17	91.84
DAM-EfficientNet	98.53	97.92	2.08	95.92

Acc is accuracy. *POD* is Probability of Detection. *FAR* is False Alarm Rate. *CSI* is Critical Success Index.

### Comparing with other state-of-the-art methods

In order to validate the reliability of the proposed DAM-EfficientNet method, this study conducted comparative experiments with other classic classification models, including AlexNet^38^, ResNet-34, DenseNet-169^39^, and ShuffleNet^40^, while keeping the training parameters consistent.

AlexNet is a well-known deep learning model with outstanding performance in classification tasks, characterized by fewer parameters and lower computational complexity. ResNet, on the other hand, is a classic model in residual neural networks, addressing the vanishing gradient problem in deep training through residual connections. The improved model in this study also utilized residual modules to avoid the issue of gradient vanishing during deep training. DenseNet is another type of dense connection network, promoting information propagation and reuse through connections with all preceding layers, aiding in the extraction of richer feature information. It was chosen as another excellent network for comparison. Lastly, ShuffleNet, like EfficientNet, is a lightweight network that reduces parameters and computational complexity while maintaining good performance through channel shuffling and group convolution. The comparison with ShuffleNet was chosen due to its smaller model size and lower computational complexity, making it suitable for experiments in resource-limited scenarios. The experimental results are shown in Table 4.

As seen from the results, DAM-EfficientNet achieved the best performance in terms of accuracy, POD, FAR, and CSI on the test set, exhibiting significant advantages over all baseline models. This indicates the effective performance of DAM-EfficientNet. ShuffleNet, with the smallest parameter size of 8.69M and FLOPs of 0.156G, had a relatively lower accuracy on the test set at 92.65%. Compared to ShuffleNet, ResNet-34 and AlexNet performed better on the test set with accuracies of 95.59% and 94.12%, respectively, indicating that increasing the depth of the model can improve accuracy. DenseNet-169 performed well in various metrics, with a parameter size (32.8M) similar to DAM-EfficientNet, but it had relatively higher FLOPs (2.88G). In practical applications, recognition accuracy is the most critical metric, and thus, DAM-EfficientNet still holds an advantage.

**Figure 8:**
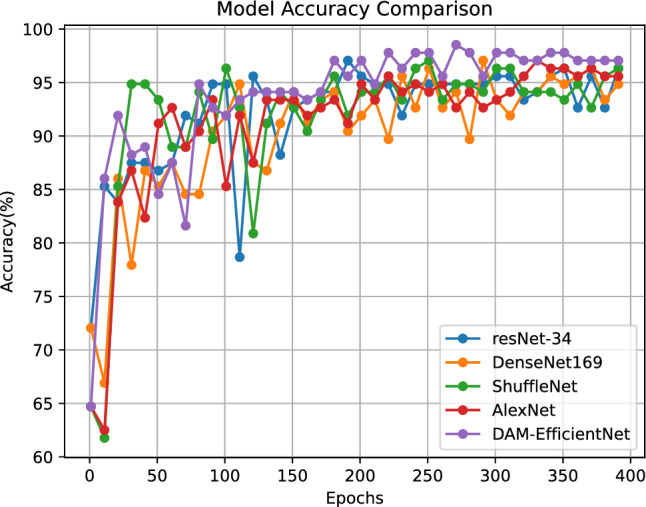
Validation accuracy curves of comparison models.

In this study, when the methodology described was benchmarked against advanced techniques on a dataset dedicated to hail classification, the proposed DAM-EfficientNet model delivered remarkable outcomes. The incorporation of spatial and channel attention modules could further refine its efficacy. This study’s algorithm exhibited notable generalization performance and optimal accuracy on the test dataset. Furthermore, DAM-EfficientNet exhibited fewer parameters and lower FLOPs compared to other baseline models. The model size was 25.46MB, only slightly larger than the lightweight ShuffleNet network. To facilitate a more intuitive performance comparison, recognition accuracy curves for each model were plotted (see Fig. 8). From Fig. 8, it can be observed that, during the training process, the convergence becomes stable around epoch 300, with less fluctuation in the accuracy of other methods. DAM-EfficientNet consistently outperforms other recognition models in accuracy throughout the training process.

**Table 4:** Validation accuracy curves of comparison models.

Model	Acc (%)	POD (%)	FAR (%)	CSI (%)	Params	FLOPs
AlexNet	94.12	89.58	6.52	84.31	61M	0.9G
ResNet-34	95.59	91.67	4.35	88.00	83.15M	3.67G
DenseNet-169	93.38	91.67	10.20	83.02	32.8M	2.88G
ShuffleNet	92.65	91.67	12.00	81.48	8.69M	0.156G
DAM-EfficientNet	98.53	97.92	2.08	95.92	25.46M	0.60G

Acc is accuracy. *POD* is Probability of Detection.*FAR* is False Alarm Rate. *CSI* is Critical Success Index. *Params* is parameters. *FLOPs* is floating point operations. *Pre* is precision.

### Verification results

**Figure 9:**
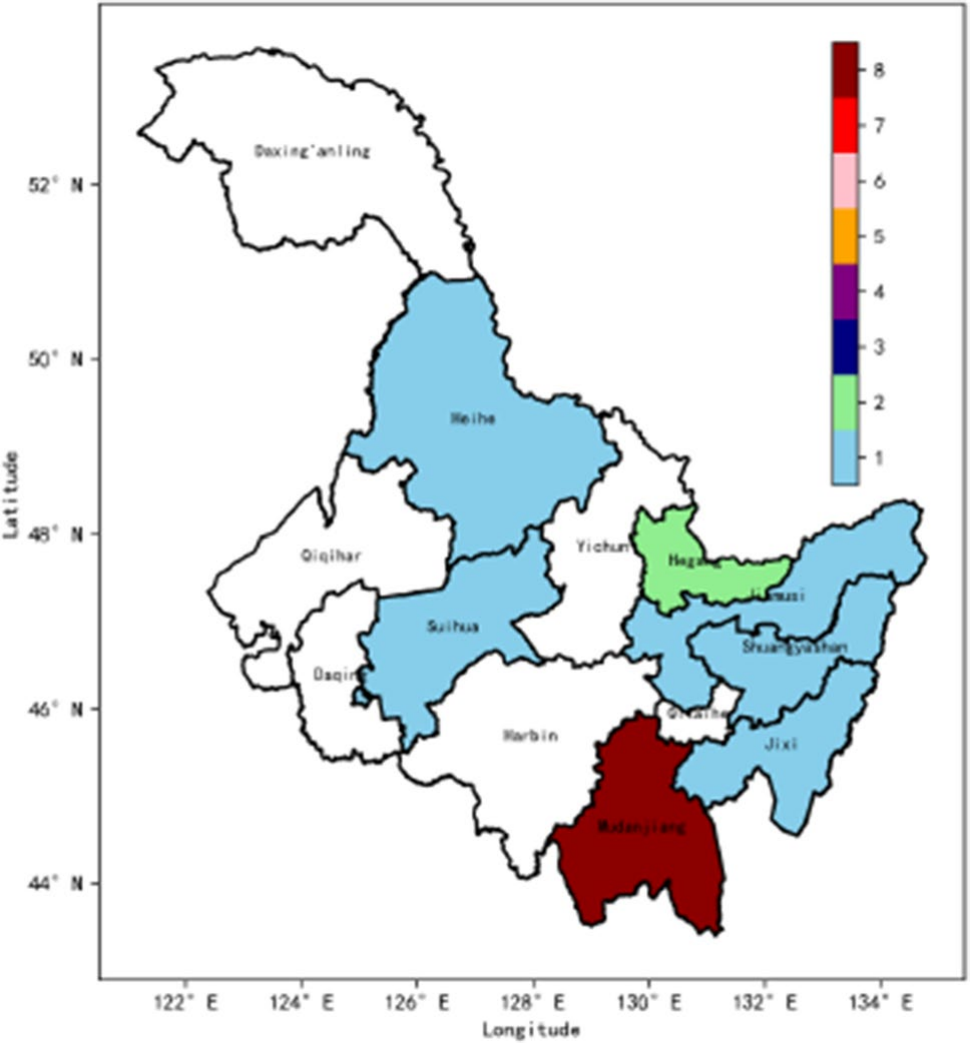
Hail Prediction Map for China’s Heilongjiang Province on June 6, 2023, from 11:15 to 11:30 The weights range from 1 to 8, representing the accuracy weight of hail prediction, with higher weights indicating more reliable results. *Note*: This map was created utilizing Python 3.8, enhanced by the crucial functionalities of Geopandas 0.8.1, Matplotlib 3.3.1, and Pandas 1.1.3. For additional information, visit: https://bit.ly/486tfYA.

**Figure 10:**
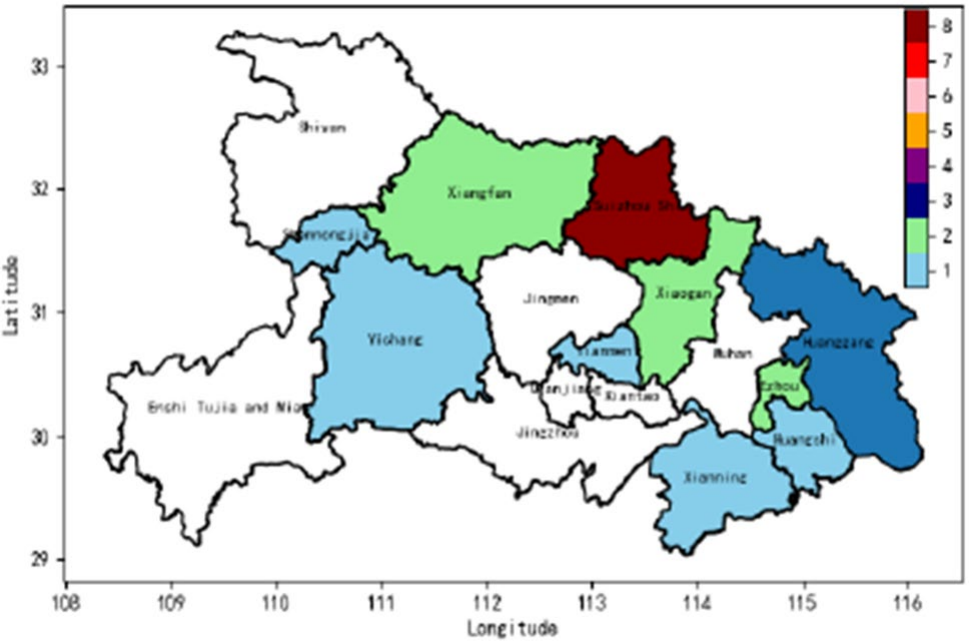
Hail Prediction Map for China’s Hubei Province on June 11, 2023, from 13:45 to 14:00 The weights range from 1 to 8, representing the accuracy weight of hail prediction, with higher weights indicating more reliable results. *Note*: This map was created utilizing Python 3.8, enhanced by the crucial functionalities of Geopandas 0.8.1, Matplotlib 3.3.1, and Pandas 1.1.3. For additional information, visit: https://bit.ly/486tfYA.

In this study, we conducted further validation of the accuracy and reliability of hail weather identification using the DAM-EfficientNet method on two hail weather events in the Heilongjiang and Hubei regions, which occurred on June 6, 2023, and June 11, 2023, respectively. Based on real-time weather conditions, hail-dominant weather was observed in Hegang, Heilongjiang, between 10:50 and 11:20 on June 6, and in Huantan Town, Suizhou City, Hubei, around 14:00 on June 11. The identification results for these specific time periods are presented in Figs. 9 and 10, respectively, where the weights ranging from 1 to 8 represent the accuracy of hail precipitation detection, with higher weights indicating more reliable results.

We performed hail weather identification on Fengyun-4 satellite imagery using channels 7 to 14 simultaneously and visualized the results as heatmaps. The heatmap’s weight indicates the number of channels in which hail was detected in a particular region, with weights ranging from 0 (no hail detected) to 8 (hail detected in all channels). The colors in the heatmap correspond to the weight value, with higher values indicating a higher likelihood of hail weather in the respective regions.

The results demonstrate that the DAM-EfficientNet model achieved the highest accuracy in hail precipitation detection in Mudanjiang, Heilongjiang (weight of 8), and Suizhou City, Hubei (weight of 8), during the specified time periods. This successful validation indicates that these regions were the most likely to experience hail during the mentioned timeframes. Overall, the DAM-EfficientNet model effectively utilized Fengyun-4 satellite data for hail weather identification. However, it has a limitation in precisely identifying local and small-scale regions.

## Conclusion

Based on ground hail observation data from Guizhou Province between 2019 and 2022, this research conducted a comprehensive study on hail identification. By employing deep learning methods to classify and train images generated from Fengyun-4 satellite cloud map files, the study achieved a high level of accuracy in hail identification. Moreover, an improved DAM-EfficientNet network structure was proposed, which demonstrated outstanding performance in hail identification. The research findings not only complement existing achievements in the field but also provide strong support for enhancing weather disaster warning and prevention systems.

The research yielded two significant outcomes. Firstly, it achieved remarkable accuracy results through the utilization of deep learning techniques for classifying and training images generated from Fengyun-4 satellite cloud map files. Secondly, the study introduced an enhanced DAM-EfficientNet network structure that exhibited superior performance in hail identification.

In comparison to prior research, this study boasts two notable advantages in the realm of hail identification. Firstly, it solely relied on Fengyun-4 satellite cloud map data for training, thereby eliminating the need to incorporate additional meteorological data. This simplified data acquisition process offers a more practical and convenient approach for hail identification applications. Secondly, the study harnessed the improved DAM-EfficientNet network structure for hail identification, surpassing other models and significantly improving identification accuracy and robustness.Therefore, the DAM-EfficientNet model is meaningful as it only requires FY-4A satellite data to detect the occurrence of hail events.

However, despite achieving commendable results, this research does have certain limitations. Primarily, the difficulty in data acquisition led to a restricted training dataset, potentially limiting the model’s generalization capabilities. Additionally, the study focused solely on Guizhou Province data, neglecting regional variations in other provinces. To address these issues, future research can expand the dataset to include observation data from other provinces, thereby enhancing the model’s adaptability and accuracy in diverse regions. Furthermore, considering temporal data training to predict hail weather in future time periods could prove beneficial in advancing the research’s practical applications.

## Data Availability

The satellite data used in this study was sourced from the China Meteorological Science Data Center (https://data.cma.cn/). The hail data in Guizhou Province, China, was provided by the Guizhou Provincial Meteorological Bureau. We have processed and organized the dataset, and it has been uploaded to GitHub (https://github.com/dhn9132/hail_images.git) for download and use. For the code that supports the findings of this research, as per the requirements, you may obtain it by contacting the corresponding author. Please consult the corresponding author for instructions on how to access the code.
